# Management Practices Affecting Lesser Mealworm Larvae (*Alphitobius diaperinus*) Associated Microbial Community in a Broiler House and After Relocating With the Litter Into Pastureland

**DOI:** 10.3389/fmicb.2022.875930

**Published:** 2022-07-01

**Authors:** Tawni L. Crippen, Baneshwar Singh, Robin C. Anderson, Cynthia L. Sheffield

**Affiliations:** ^1^Southern Plains Agricultural Research Center, Agricultural Research Service, United States Department of Agriculture, College Station, TX, United States; ^2^Department of Forensic Science, Virginia Commonwealth University, Richmond, VA, United States

**Keywords:** darkling beetle, broiler chicken, poultry litter, arthropod, fertilizer

## Abstract

Lesser mealworms are often found infesting production houses used to raise broiler chickens. Previous studies have investigated pathogenic microorganisms associated with the larvae, but a more thorough study relating total microbiome changes due to management procedures and flock rotations was needed. Additionally, there is a question of what microbiota are transferred into the environment when the litter, in which larvae reside, is piled in pastureland for use as fertilizer and where interactions with the soil and other fauna can occur. This study chronicled, by the 16S rRNA sequencing, the bacterial community profile of larvae in a broiler grow-out house synchronizing to when birds were added to and removed from the house over 2.5 years. The profile was found to be relatively constant despite 11 flock rotations and management disruptions, specifically litter cleanout procedures and the addition of new birds or bedding. In contrast, once removed from the controlled broiler house environment and placed into open pastureland, the substantial microbial diversity brought with the larvae showed greater fluctuation in structure with environmental conditions, one of which was rainfall. Surprisingly, these larvae survived at least 19 weeks, so the potential for moving larval-associated microbes into the environment needs further assessment to minimize the risk of relocating foodborne pathogens and also to assess those bacteria-generating metabolites that have benefits to plant growth when using the litter as a fertilizer. The characterization of their microbiome is the first step to investigating the influences of their microbes on the manmade and environmental ecosystems.

## Introduction

Relatively little is known about the lesser mealworm (LM), *Alphitobius diaperinus* (Panzer) (Coleoptera: Tenebrionidae). The majority of the scientific literature on these beetles focuses on pest management procedures to eradicate the insect from stored grain silos and poultry production facilities. While it is a pest, the LM is also being explored for commercial production of insect protein for use as feed, for commercial production of frass for use as a fertilizer, and as a possible environmentally friendly method to degrade plastics (Cucini et al., 2020; Leni et al., 2020). In all arenas, more information about the microbiome carried by this arthropod is of interest to industries for food safety reasons and the retention of possible beneficial microbial populations.

The most abundant and diverse clade of animals are those of the phylum Arthropoda and each carries a vast microbiome with it (Ødegaard, 2000; Basset et al., 2012). The bacterial phyla Proteobacteria and Firmicutes usually predominate in the arthropod alimentary canal regardless of the different feeding strategies being utilized; however, at the lower taxa levels (i.e., class, order), the bacterial community composition varies widely and are key facilitators of the varied lifestyles of their arthropod hosts (Colman et al., 2012; Yun et al., 2014). Not unexpectedly, the bacterial community structure is influenced by diet and host taxonomy but is also altered by other factors, such as the developmental stage, the host niche environment, and transient biotic and abiotic environmental factors (Colman et al., 2012; Engel and Moran, 2013; Yun et al., 2014).

The LM has been found throughout the world for so long that its actual origins are uncertain, but it is thought to be native to sub-Saharan Africa. As a tropical species, it does well in warm, humid environments like caves but inconveniently also in grain silos and poultry houses. We know that LM harbor viral and bacterial pathogens (Crippen and Poole, 2012; Donoso et al., 2020; Tamburro et al., 2022), but to date, little has been done to explore the entire microbiome within its typical habitat of a broiler facility or the changes that occur over time as multiple flocks are grown out on the poultry litter of that habitat. Additionally, there is a dearth of knowledge about the microbiome of LM found within litter that is subsequently used as fertilizer for crops, gardens, and pastureland. Broiler litter contains nearly 30% crude protein, along with minerals and some heavy metals, and is considered a valuable commodity for secondary use as a fertilizer once it can no longer sustain broiler chickens (Martin and McCann, 1998; Crippen et al., 2016). Arunraj et al. (2013) demonstrated that LM in poultry litter transported and placed in open fields did not immediately die, but dispersed and hid in the local environment; although that study ended after only 10 days. It is important to understand the bacteria that come with these arthropods not only when the larvae are within the poultry house but also when they are redistributed into the environment. In that regard, the research reported here was designed to determine the microbiome of late instar larvae of the lesser mealworm (LML) from a newly constructed broiler house through 11 flock rotations (2.5 years) and after the spent litter was transported to pastureland for use as fertilizer. We also considered how management practices within the production facility affect the retention or decline of the bacteria comprising the LML microbiome.

## Materials and Methods

### Site Description

This new dirt-floored broiler production house was constructed in NW Robertson County, Texas, on an open range Post Oak Savannah (Gould et al., 1960). The land was privately owned, so permits and approvals for the work were not needed, and we had permission from the landowner to conduct this research. The USDA-NRCS Official Soil Series Description of the soil was a fine, smectite, thermic Udertic Paleustalfs with slopes ranging from 0 to 3%^1^ (accessed January 2022). A conventional tunnel ventilated metal broiler facility, 14 m wide (North/South) by 152.4 m in length (East/West) in size, placed on 25 cm of commercial-grade clay-based topsoil was sampled. Alternating water and feed lines ran the entire East/West length of the house (Supplementary Figure 1). Eleven healthy flocks were grown out in the location from February 2008 to August 2010. Each flock had an average duration of 59 ± 6 days and the house was left unoccupied for an average of 11 ± 5 days between flock rotations. No other sites were visited on the same day as collection from this site and full personal protective equipment for aseptic technique were used for personnel and equipment throughout the collection.

### In-House Management Practices

The temperature-controlled production house had approximately 32 metric tons (15.3 cm in depth) of fresh pine chip bedding (PCB) added to the floor prior to the arrival of the first flock. The broiler chickens (Ross^®^ 708) at 1 day of age were stocked at a density of one broiler per 0.1 m^2^ (25,800 birds per rotation) and fed a corn/soy-based ration with bacitracin (50 ppm), a product of *Bacillus licheniformis* (see the regime in Supplementary Table 1). Bacitracin has bactericidal activity against some Gram-positive organisms and suppresses necrotizing enteritis in poultry^2^ (accessed January 2022). The chickens were reared from 1 to 2 days of age through 6–9 weeks of age; averaging 59 days of rearing with 11 days empty between flock rotations. Litter is defined as bedding after use by the birds; therefore, litter consists of bedding with chicken manure, urine, carrion, feathers, insects, spilled feed, and any other materials deposited during the grow-out period. After the seventh flock rotation, a partial house cleanout (PCO) was performed, which entailed the removal of the top cake layer of litter and hardened manure (5–8 cm). Fresh PCB (6.4 cm) was then added to the house. A total house cleanout (TCO) was performed after the ninth flock rotation, which removed all litter plus 1–3 cm of the pad-soil. Fresh PCB (15.3 cm) was added to the house prior to placing the 10th flock.

### Collections of Larvae Within the Poultry House

Birds were first placed into the new house on the third week after construction was completed. The house was divided into Side A and Side B for sampling because birds were confined to half of the house (Side B) for the first 2 weeks of each flock rotation by a brooder curtain before being allowed access to the entire house. The house was divided into 10 collections sites (Supplementary Figure 1), and collections were attempted weekly during the first flock rotation. Late instar LML ranging from 7 to 11 mm in length were collected and the LML of sufficient length were first found in the final week (week 7) of the first grow-out period (Flock 1) and to be initially populated along the inside perimeter of the building. It is unknown how they were introduced into the house. Flocks 2 and 4–11 were sampled only during the first and final weeks of each flock rotation (Flock) within 24 h after bird placement or bird removal. Flock 3 was not sampled owing to logistical considerations with the producer and national holidays. The larvae populations slowly expanded to the center of the facility, mostly hiding under the feeders until they finally populated the entire house. Thus, the larval collections were designed at each time point based on larval locations. At the onset, 12 replicate 7.6 × 30.8 cm polyvinyl chloride pipe tubing traps with rolled fluted corrugated cardboard inserts were placed along the perimeter of the house in areas 1, 5, 6, and 10 as seen in Supplementary Figure 1. For collection, each corrugated insert was sterilely removed, and larvae were shaken into individual resealable sterile plastic bags. This method was continued for the 5 collection time points between weeks 7 and 42 (the end of Flock 4) (*n* = 60). At the start of Flock 5 (week 43), the LML had spread from the perimeter to the interior of the structure and were collected from under the feeder stations along the three feeder lines: areas 2, 3, 4, 7, 8, and 9 as seen in Supplementary Figure 1 covering each side (A or B). The samples were collected using sterile gloves and shovels cleaned with alcohol wipes between each sampling location. A total of three random sites within each of the six interior areas were sampled (*n* = 252) along with the 12 perimeter samples (*n* = 168) over the remaining 14-time points. All samples were transported from the field site at ambient temperature and were processed the same day upon return to the laboratory. The perimeter and interior samples were combined at the laboratory into 2 composite samples per side A (areas 1, 2, 3, 4, and 5) and 2 composite samples per side B (areas 6, 7, 8, 9, and 10) per each time point. A total of 480 samples were collected in-house over the course of the study and combined into 76 composite samples for DNA analyses (In-House Larvae).

### Collections of Larvae in the Spent Litter

The litter was removed (21 MT/truck) during the TCO after Flock 9 and transported approximately 30 km to pastureland. A composite sample of the soil in the pastureland was taken at the location where the litter was eventually piled (Native soil). The pastureland had not previously received litter supplement. Litter (5.5 truckloads) was placed in 6 contiguous rows ranging in height from 1.8 to 2 m; covering approximately 2,100 m^2^ of surface area. A single composite larval sample (Spent Larvae), collected at each time point (0, 1, 2, 3, 5, 7, 9, 11, 13, 15, 17, and 19 weeks), was made from each of the 10 random sites across the pile from initial deposition in April until spreading in August. Spreading onto the pastureland at a rate of 0.9 MT/0.4 ha occurred between weeks 19 and 20. Two days after spreading, no larvae were found at the site, so a combined soil and litter sample was taken (Post Spread); no LML could be found at the site after the spreading. Weekly weather data were gathered from Weather Underground, an online service of The Weather Company, Inc., Atlanta, GA, United States a network of 140,000+ NOAA and personal weather stations which provide the best locally based weather data points (Supplementary Table 2). All samples were placed into sterile resealable plastic bags and kept at ambient temperature during transport to the laboratory. The LML was separated from the litter using sterile forceps and processed the same day. A total of 120 Spent Larvae samples were collected from the site over the course of the study and combined into 12 composite samples for DNA analyses.

### 16S rDNA Analysis

In the laboratory, all larvae from each area were separated from their substrate using sterile forceps and collection trays. Two aliquots of 30 larvae from each area were prepared. One sample was surface disinfected as previously described (Crippen and Sheffield, 2006) and the other was processed without surface disinfection. The samples were homogenized in a lysing solution from the FastDNA™ SPIN Kit (MP Biomedicals, Santa Ana, CA, United States) and then incubated at 4° overnight before DNA was extracted per manufacturer instructions. The DNA was quantified using the Quant-iT™ PicoGreen™ dsDNA Assay Kit (Thermo Fisher Scientific, Waltham, MA, United States) and a Wallac 1420 Victor 3™ fluorescent microplate reader (PerkinElmer, Waltham, MA, United States). The DNA concentration was standardized to 20 ng/μl and area samples were combined into 4 composite larval samples (as described above) from within the house (In-House Larvae) and Spent Larvae per time point. The samples were thoroughly mixed by slow rotation for 5 min. The resulting extracts were sent overnight to the Research and Testing Laboratory, LLC. (Lubbock, TX, United States) for 16S rDNA sequencing as previously described (Crippen et al., 2021). Suspected chimeric sequences were deleted and the rest good quality sequence reads (169,892) were utilized for hierarchical classifications previously described (Crippen et al., 2021). Only sequences having ≥ 80% bootstrap support were considered classified at a particular hierarchical level. Rarefaction curves were generated at 3 and 10% genetic distances for both In-House and Spent Larvae datasets (Supplementary Figure 2). To avoid spurious OTU counts because of the different number of sequence reads in different samples, all sequences were subsampled (to 542 reads for In-House and 581 reads for Spent Larvae samples, respectively) for α-diversity (Inverse Simpson and Shannon diversities at 0.03 and 0.1 genetic distances) and β-diversity (Yue & Clayton at 0.03 genetic distance) estimations. Sub-sampling caused a loss of 8 samples out of a total of 91 composite samples from further analysis. Yue and Clayton distances were utilized for the non-metric multidimensional scaling (NMDS) plot in Mothur v 1.41.3 and data from the first three axes for all treatments were plotted using the rgl v 0.100.54 package in R version 4 (Language and Environment for Statistical Computing, 2011).

Using natural log-transformed percent relative sequence abundance profiles where 0% values were converted into 0.01%, heat maps graphics were generated from different treatments in the gplots v 3.0.3 package of R version 4 and in Prism 7 (Graph Pad, La Jolla, CA, United States). Bacterial genera associated with In-House and Spent Larvae samples on the x-axis were clustered by Unweighted Pair Group Method with Arithmetic Mean (UPGMA) based on weighted Unifrac distances. All trees were edited using FigTree v1.4.2^3^ (accessed January 2022). All raw sequence files were submitted to European Nucleotide Archive Database as part of the study (accession# PRJEB47987).

### Statistical Analysis

Preliminary analysis of molecular variance (AMOVA) using Yue and Clayton distances (at 3% genetic dissimilarity) demonstrated no significant differences within flock rotations or between collection sites (Side A versus Side B) and no significant difference in surface disinfected and non-disinfected larvae (Supplementary Figure 3). Therefore, these samples were combined as replicates based on flock rotation for further analysis. Bacterial genera associated with In-House and Spent Larval samples were clustered by Unweighted Pair Group Method with Arithmetic Mean (UPGMA) based on weighted Unifrac distances. All trees were edited using FigTree v1.4.2 (see footnote 3). Indicator genera associated with each treatment were determined using package “indicspecies” version 1.7.9 (De Cáceres et al., 2011) in R version 4 (Language and Environment for Statistical Computing, 2011). The datasets generated for this study were deposited in the European Nucleotide Archive database as part of the study (accession# PRJEB47987).

## Results and Discussion

### In-House Larvae

Larval samples were collected from this newly built broiler house through the partial cleanout (after Flock 7), the total cleanout (after Flock 9), and the eleventh flock rotation to investigate the microbial diversity and community changes within the LML population. A low level of bacitracin was used in the feed for these birds; it has bactericidal activity against some Gram-positive organisms and suppresses necrotizing enteritis in poultry and could have effects on the microbial diversity within the house. Originally, the larvae were collected from each site as two groups in which one group was surface disinfected and the other was not prior to homogenization for DNA extraction. It was determined that the microbial profiles of the two groups were not significantly different (Supplementary Figure 3). Therefore, the results here reflect the microbial community of the whole insect as it would exist in nature. It should be pointed out that the sequencing platform only sequences small fragments, which when trying to identify bacteria at the species level is less precise; therefore, we only discussed taxa at the genus level or higher.

#### In-House Larvae: Alpha Diversity

The bacterial taxonomic diversity between the successive flock rotations in the house LML was investigated (Table 1) and the corresponding rarefaction curves are shown in Supplementary Figure 2. The rarity and commonness of species present in the bacterial communities associated with LML are shown by the indices in Table 1. In considering both the number of unique microorganisms (richness) and relative abundance of different species present (evenness), with emphasis on the evenness component, the Inverse Simpsons index measured higher values, indicating having the most diverse community for larvae from Flock 1, while Flock 9 larvae had the lowest diversity of microbial organisms. LML from Flock 1 had the most diverse microbial population and Flock 9 larvae had the least diverse population as indicated by the Shannon index, which emphasizes the richness. Overall, the diversity of microbes associated with the LML that entered the house at the end of Flock 1 decreased in subsequent flock rotations once the arthropods were in the poultry house.

**TABLE 1:** The coverage, diversity, and evenness indices at 0.03 and 0.10 genetic distances of bacteria from In-House *A. diaperinus larvae* associated with flock rotations.

	Coverage		Inverse Simpson Index		Shannon Index		Shannon Evenness Index	
In-House	0.03	0.1	0.03	0.1	0.03	0.1	0.03	0.1
Flock 1	0.93	0.96	18.80	10.69	3.53	2.97	0.80	0.74
Flock 2	0.94	0.96	4.65	4.74	2.39	2.20	0.57	0.57
Flock 4	0.95	0.97	10.99	7.46	2.56	2.29	0.60	0.59
Flock 5	0.92	0.95	11.21	9.44	3.01	2.76	0.67	0.66
Flock 6	0.91	0.95	13.76	9.35	2.64	2.34	0.58	0.56
Flock 7	0.92	0.95	9.32	7.90	2.84	2.55	0.64	0.63
Flock 8	0.93	0.96	6.31	5.85	2.61	2.39	0.62	0.62
Flock 9	0.93	0.96	3.46	3.34	2.16	1.93	0.51	0.50
Flock 10	0.90	0.95	9.49	8.20	3.02	2.65	0.67	0.65
Flock 11	0.91	0.95	9.90	8.06	3.02	2.68	0.66	0.65

It is of interest to know if the management practices and the microbiome of the birds at the facility influenced the retention of beneficial and harmful microbes within the LML population. In that regard, the cleanout procedures and the addition of new bedding after cleanout for Flocks 8 and 10 resulted in no consistent changes in the microbial diversity within the larvae, decreasing in Flock 8 but increasing in Flock 10 from the prior flock. However, the microbial community within each broiler house is influenced by many factors, such as the type of bedding and constituents of the feed ration; the breed, microbiome, and age of birds reared; the amount of water and feed spilled; the number of birds stocked per house; and the number of times the poultry house is cleaned (Chastain et al., 2021). These factors vary by production facility and their standard operating procedures usually conforming to the particular poultry industry for which the birds are being produced; therefore, this result may not be universal for all broiler houses.

#### In-House Larvae: Indicator Species

Indicator species analysis for the In-House larval samples identified 14 indicators that had notably increased in percent abundance during the different flock rotations as designated (Table 2) and each of these bacteria has been previously reported to be associated with poultry and/or insects in beneficial or pathogenic modes. The top three indicator species include *Brevibacterium*, usually a soil organism, but has also been found on the skin and marine fish as well as having been isolated from poultry and poultry litter (Dumas et al., 2011; Gilroy et al., 2021). It can utilize ammonia and ammonium sulfate and reduce nitrates (NO^–^_3_) to nitrites (NO^–^_2_). Ammonia is a highly irritating gas formed through the microbial decomposition of uric acid, which is excreted by the birds, so a reduction in ammonia emissions is beneficial. Sphingomonads are distributed widely in the environment in soil and water and are considered important biocatalysts for soil bioremediation, but they also display opportunistic pathogenicity in immunocompromised humans but not nonhuman animals (Yabuuchi and Kosako, 2005). Sphingomonads are very beneficial nutrient cycling bacteria that can also use recalcitrant polycyclic aromatic hydrocarbons as their sole carbon source and possess unique abilities to degrade a variety of pollutants, including azo dyes, chlorinated phenols, dibenzofurans, insecticides, and herbicides (Leys et al., 2004). As such this bacterium appears to play a detoxifying role in the arthropod gut microbiome (Jing et al., 2020). They secrete sphingan polysaccharides and some strains are also nitrogen-fixing bacteria (Videira et al., 2009). A Sphingomonad that was isolated from a chicken cecum and then fed to chickens was able to decrease ammonia in the resulting chicken manure (Wang et al., 2021). *Jeotgalicoccus*, a genus within the Staphylococcaceae family, is also widely distributed in nature. *Jeotgalicoccus* sp. have been previously found in poultry houses isolated from the feed supplements (*J. coquinae*), the air filter system of a turkey house (*J. areolatus*), and pig barns (*J. schoeneichii*) (Martin et al., 2011; Glaeser et al., 2016). Yet to be defined bacteria (unclassified) were also indicator species.

**TABLE 2:** Indicator species for In-House *larvae* sampled during flock rotations.

Genera	Indicator Group	PBCC*
*Brevibacterium*	Flocks 1, 11	0.725
*Sphingomonas*	Flocks 4, 5	0.716
*Jeotgalicoccus*	Flock 1	0.677
Bacteria_unclassified	Flock 9	0.638
*Gordonia*	Flock 10	0.559
*Facklamia*	Flock 1	0.551
*Paenibacillus*	Flock 6	0.547
*Corynebacterium*	Flocks 1, 7, 10	0.546
*Luteimonas*	Flock 11	0.534
Bacillales_unclassified	Flocks 1, 2, 4	0.529
*Dietzia*	Flocks 1, 10, 11	0.526
*Yaniella*	Flocks 7, 8, 9	0.506
*Alkalibacterium*	Flock 6	0.505
*Halomonas*	Flocks 6, 11	0.501

Only those taxa whose relative abundances across all flocks were 0.05% or higher and had a p-value of ≤ 0.01 and Point Biserial Correlation Coefficient (PBCC)* value ≥ 0.50 are shown.

*PBCC: Is a correlation between two binary vectors, similar to Indicator values. The higher the PBCC values, the stronger the likelihood that a particular taxon is an indicator of that sample.

The next group of indicator species all had point biserial correlation coefficients below 0.6. *Gordonia*, identified in 1997 (Stackebrandt et al., 1997), are found in aquatic and terrestrial habitats and are Actinomycetes that contain unique long-chain fatty acids (mycolic acids) in the lipid-rich cell walls and can reduce nitrates. Like Sphingomonads, *Gordonia* also has the ability to degrade environmental pollutants, such as polycyclic aromatic hydrocarbons, alkylpyridines, and phthalates; triazines xenobiotic compounds and natural polymers, such as rubber; and xenobiotic compounds (e.g., 1,3,5-triazines such as RDX) (Arenskotter et al., 2004). However, they are also opportunistic pathogens. *Facklamia* are from the lactic acid bacterial family of Aerococcaceae, associated with environmental sources and are warm-blooded animals (Hoyles, 2014). They have been identified in poultry feed and as one of a group of bacterial taxa that correlated positively or consistently with the performance of young birds (Johnson et al., 2018; Haberecht et al., 2020). *Paenibacillus* inhabit a wide range of environments, and they have been isolated from corn silage (Tohno et al., 2016). Its ability to promote plant growth through nitrogen fixation, phosphate stabilization, production of phytohormones, and enabling iron acquisition along with its production of antimicrobials, enzymes, and other molecules makes this species of interest to industry (Grady et al., 2016). When used as a dietary supplement, it improved broiler performance and reduced cecal *Escherichia coli* load (Ekim et al., 2020).

*Corynebacteria* are found in the microbiota of many animals, including poultry, and mostly exist commensally (Kraft et al., 1966). However, some can be opportunistic pathogens, such as *Corynebacterium diphtheriae*, the causative agent of diphtheria, which was found in a diphtheria outbreak in broiler chickens (Lu et al., 2016). The pathogen *Corynebacterium pseudotuberculosis* can be mechanically transmitted by house flies (*Musca domestica L*.). No information exists about its association with *Alphitobius diaperinius*, but it is also one of a group of bacterial taxa that correlated positively or consistently with the performance of young birds (Barba et al., 2015; Johnson et al., 2018). *Luteimonas* are common in soil and marine sediments. It has been described in the gut of poultry and isolated from the wormcast of the earthworm (*Eisenia foetida*, Savigny) (Cha et al., 2020; Gilroy et al., 2021). *Dietzia* spp. are widely distributed in nature and well-known for synthesizing carotenoids and have been used as a source of canthaxanthin since vertebrates cannot synthesize carotenoids *de novo* and rely on dietary sources of intake (Tao et al., 2007; Koerner et al., 2009). It has been found to correlate positively or consistently with the performance of young birds (Johnson et al., 2018). *Yaniella* was originally isolated from soil, has been found in poultry litter, and is capable of nitrate reduction and H_2_S production (Chen et al., 2010; Dumas et al., 2011; Wang et al., 2016). Both *Halomonas* and *Alkalibacterium* species are halotolerant, alkaliphilic bacteria, widely distributed due to their ability to grow in harsh saline environments. Alkalibacteria is a lactic acid bacteria (Lawson and Caldwell, 2014). *Halomonas* sp. have been found in insect gut microbiomes and have cellulolytic activity *via* the production of cellulase and are thus a component of the carbon cycling process in the environment (Shivanand et al., 2013; Xu et al., 2018). *Halomonas* has been noted to decrease nitrite and nitrate during manure composting (Zainudin et al., 2020).

#### In-House Larvae: Relative Abundance at the Phylum Level

**FIGURE 1:**
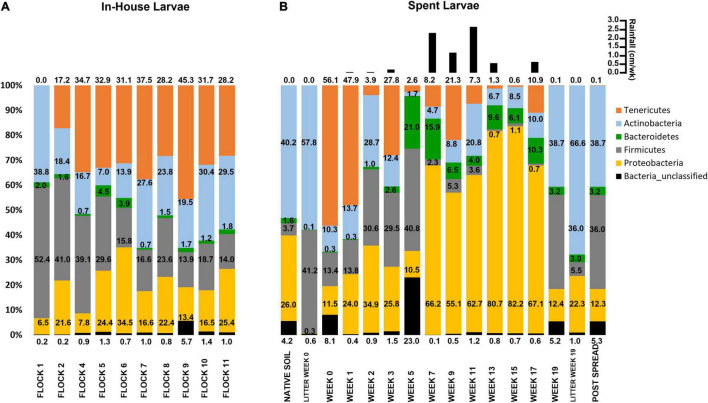
Histograms showing the mean relative abundances of bacterial phyla associated with **(A)** In-House Larvae samples taken during successive flock rotations (1 through 11), and **(B)** Spent Larvae taken after deposition of the spent litter into pastureland, including pastureland soil prior to deposition of the spent litter (Native Soil), samples of the spent litter at the time of deposition onto the pastureland (Litter Week 0) and just prior to spreading (Litter Week 19); the soil/litter combination after spreading onto the pastureland (Post Spread); and the larvae collected from that litter over the 19 weeks of stockpiling (Week 0 through Week 19). The values correspond to the percentage of total sequences of that taxon for that sample. In the upper right-hand corner is weekly total rainfall (cm/week) for Weeks 1–3 and Post Spread and bi-weekly total rainfall for Weeks 3–19 at the site until the litter was spread on the field.

The most abundant phylum (28.7%) associated with the larvae is a group of very small, difficult-to-detect bacteria, the Tenericutes (Brown, 2010). The LML arrived at the poultry house during the final week of sampling the first flock rotation. Tenericutes were found in the larvae in Flocks 2 through 11 when the larvae were present in abundance. These bacteria do not synthesize a peptidoglycan cell wall and are bound only by a plasma membrane. They consist of the class Mollicutes with genera that tend to live intracellularly or intercellularly as parasites, (endo)symbionts, pathogens, or commensals. Tenericutes are a distant ancestor to the next most abundant phylum of bacteria, the Firmicutes (26.5%), which are mostly thick peptidoglycan cell walled, endospore-producing bacteria. This phylum consists of two major groups with both beneficial and pathogenic members: the anaerobic *Clostridia* that ferment carbohydrates in the gut producing short-chained fatty acids and the Bacilli which are diverse obligate or facultative aerobes (Madigan et al., 2012). The Actinobacteria were also abundant (22.6%) and are mostly aerobic bacteria often found in the soil, but some are aquatic. They perform a wide variety of metabolic processes fundamental to decomposition and humus formation (Ventura et al., 2007). Accordingly, they have great economic importance due to their positive effects on soil ecosystems; additionally, many of their secondary metabolites have antibiotic qualities. Proteobacteria are the fourth most abundant (18.9%) phylum and represent a very diverse group of bacteria that perform a wide variety of metabolic processes. This phylum includes a variety of foodborne pathogens important to the broiler production industry, notably *Salmonella* sp. and *Escherichia coli*. Besides some unclassified bacteria (1.3%), Bacteroidetes were the least represented (1.9%). These bacteria have both anaerobic and aerobic non-spore-forming species that are ubiquitous in the environment. Some are symbiotic species in animals where they perform the crucial function of degrading proteins and complex sugar polymers for their hosts. Overall, the abundance of Firmicutes associated with the larvae fluctuated the most (26.4 ± 13.5%) and appreciably decreased over the successive flock rotations, seeming to stabilize by Flock 6 through 11 (17.1 ± 3.6%). However, overall, the bacterial community structure associated with LML dwelling within the house remained relatively stable during Flocks 2 through 11 (Figure 1A).

Yun et al. (2014) reported combined results of gut-associated bacteria from 218 various species of insects using primers targeting the V1-V2 region of the 16S rRNA. Their results showed that the general insect gut microbiota was dominated by Proteobacteria (62.1%) and Firmicutes (20.7%). While our results represent both external and internal bacteria using primers targeting the V1-V3 (27F/519R) region of the 16s rRNA gene, other studies targeted different variable regions while investigating LM and the closely related yellow mealworm larvae (YM; *T. molitor*) larvae under different sampling conditions (Supplementary Table 3). The taxa differences demonstrate the variability of bacteria carried by LM and YM larvae dependent on their biotic and abiotic surroundings. Briefly, Jung et al. (2014) also targeted the V1–V3 (27F/518R) region in an analysis of the YM purchased from a local market and found a community dominated by Tenericutes (36.3%) and Proteobacteria (34.1%). Using shorter primers targeting the V3 (338F/518R) region, Garofalo et al. (2017) measured the microbiome of marketed, dried *T. molitor* larvae and also found them dominated by Tenericutes (44.2%) and Proteobacteria (39.2%). Cucini et al. (2020) used primers targeting the V3–V4 region (314F/805R) and determined that the microbiome of larvae artificially reared on a factory protocol diet was dominated by Proteobacteria (57%) and Bacteroidetes (26%). Using primers targeting the V4 region (515F/806R), Wynants et al. (2018) measured the microbiome in the LM larval stages (28–36 days) and found a predominance of Proteobacteria (68–78%), followed by Firmicutes (∼20%). Using primers targeting the V4–V5 (577F/926R) region, Stoops et al. (2017) analyzed YM larvae and also found a community dominated by Proteobacteria (35.9%) and Firmicutes (31.3%).

Our study on the In-House LML showed a lower percentage of Proteobacteria and a higher percentage of Tenericutes and Actinobacteria than the other studies on the LM, which could have resulted from using different primers and analysis pipelines, as well as from the rearing environment and diet (Colman et al., 2012; Abellan-Schneyder et al., 2021). Our previous study (Crippen et al., 2021) of the microbial profile of the In-House litter in which this LML dwelt demonstrated a consistent increase in the proportion of Actinobacteria and a decrease in Firmicutes over the 11 flock rotations, despite partial or total cleanouts. The soil bacterial ecosystem under this litter, which the LML inhabited, likewise showed an increase in Actinobacteria with successive flock rotations (Crippen et al., 2019). In the litter, Proteobacteria had a slow decline with a slight recovery after the partial and total cleanouts and the addition of new bedding (Crippen et al., 2021). Comparatively, the LML also showed a consistent decrease in the proportion of Firmicutes over the 11 flock rotations. However, the average proportion of Actinobacteria was less and Proteobacteria was more than what was present in the litter. Additionally, the larva carried 17–45% Tenericutes.

#### In-House Larvae: Beta Diversity and Analysis of Molecular Variance

**FIGURE 2:**
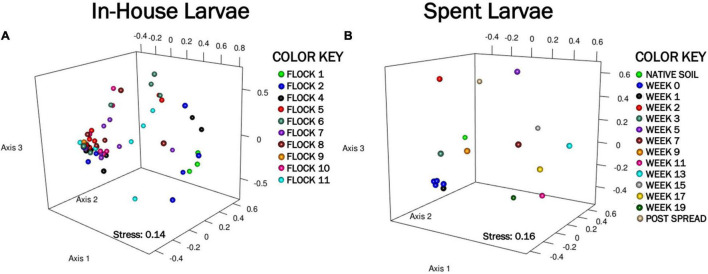
A nonmetric multidimensional scaling (NMDS) ordination representing the bacterial community composition associated with larvae, generated using Yue and Clayton distances, shows dissimilarities between components. **(A)** Shows clustering of the In-House Larvae (stress = 0.14, *r*^2^ = 0) over 11 flock rotations. **(B)** Shows clustering of the Spent Larvae (stress = 0.16, *r*^2^ = 0) through 19 weeks after piling in pastureland.

**FIGURE 3:**
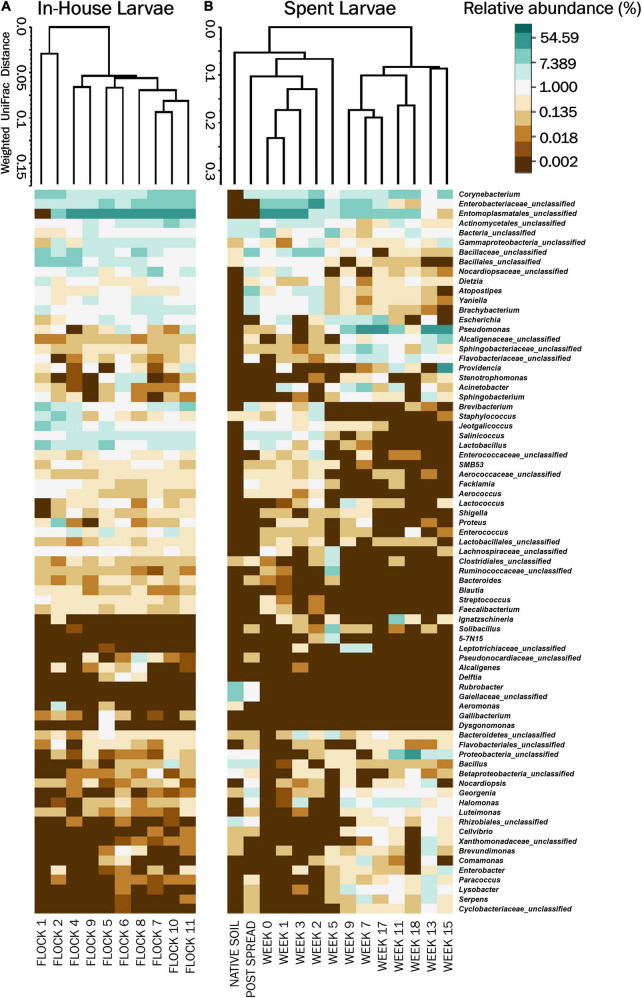
Heatmaps of the percent relative abundance of the top 75 bacterial genera associated with LML, **(A)** in the poultry house during the flock rotations (In-House Larvae); and **(B)** subsequent to the pastureland stockpiling of the spent litter from the house (Spent Larvae). Also included is the microbiome of the soil prior to the placement of the litter and LML into the field (Native Soil) and after the spreading of the litter and LML onto the pastureland (Post Spread). Samples were clustered using Weighted Pair Group Method with Arithmetic Mean (UPGMA) tree based on weighted Unifrac distances. For natural log transformation “0” was converted to “0.01.”

The non-metric multidimensional scaling (NMDS) plot, generated using Yue and Clayton genetic distances, showed very few dissimilarity patterns between components (Figure 2A). There was an overlap in the bacterial community structures among the larvae with the exception of Flocks 1 and 2 separating from later flocks. The weighted heat map of phylogenetic differences in the bacterial structure at the genus level similarly showed a separation of Flocks 1 and 2 into their own clade and then very little change in microbial profiles between flocks for subsequent rotations (Figure 3A). Using AMOVA analyses of OTUs at the genus level shows that only Flocks 1 and 6 are significantly different (*p* ≥ 0.05). The major management practice disruption of a partial (after Flock 7) or a total (after Flock 9) clean out seemed not to disturb the microbial community associated with the LML (Figure 3A).

### Spent Larvae

The Spent Larvae of LM were collected from the litter taken from the total cleanout of the poultry house (after Flock 9) that was piled onto pastureland. During this study, LML were found alive within the poultry litter in the pasture until it was spread as fertilizer during week 20, after which no beetles could be located at the pile site. While in the pastureland, the larvae were exposed to all of the biotic and abiotic environmental fluctuations common to the location, including local fauna, intermittent rainfall, and temperature fluctuations.

#### Spent Larvae: Alpha Diversity

The bacterial taxonomic diversity in the Spent Larvae samples was investigated (Table 3) and corresponding rarefaction curves are shown (Supplementary Figure 2). The larvae surviving in the spent litter piles over the weeks until it was spread showed a large range in microbial diversity that did not seem to stabilize while in the pastureland. The Inverse Simpsons at 0.01 genetic distance ranged from 3.3 during week 0, which had the highest proportion of Tenericutes; to 74.7 during week 5, which showed a spike in unclassified bacteria, Firmicutes, and Bacteroidetes, and a decrease in Tenericutes, Actinobacteria, and Proteobacteria from the previous weeks. This variability in diversity did not appear to correlate with rainfall or temperature changes at the site (Supplementary Table 2) or with microbial diversity changes in litter samples taken from the site (Crippen et al., 2021).

**TABLE 3:** The coverage, diversity, and evenness indices at 0.03 and 0.10 genetic distances of bacteria associated with *A. diaperinus larvae* from the Spent litter the weeks after it was placed into the pastureland.

	Coverage		Inverse Simpson Index		Shannon Index		Shannon Evenness Index	
Field	0.03	0.1	0.03	0.1	0.03	0.1	0.03	0.1
Week 0	0.93	0.96	3.46	3.33	2.18	1.92	0.51	0.50
Week 1	0.92	0.95	4.15	3.86	2.61	2.26	0.59	0.55
Week 2	0.90	0.95	24.30	10.15	3.82	3.15	0.81	0.74
Week 3	0.90	0.94	8.99	7.80	3.30	2.94	0.70	0.68
Week 5	0.45	0.70	229.90	74.73	5.68	4.97	0.95	0.89
Week 7	0.92	0.96	23.60	7.76	3.56	2.69	0.79	0.68
Week 9	0.89	0.95	12.58	8.81	3.37	2.77	0.72	0.67
Week 11	0.86	0.92	18.83	10.61	3.61	2.98	0.75	0.68
Week 13	0.89	0.95	13.22	3.97	3.53	2.46	0.74	0.59
Week 15	0.88	0.92	6.45	5.50	2.83	2.48	0.61	0.58
Week 17	0.88	0.94	17.94	5.88	3.53	2.56	0.75	0.61
Week 19	0.87	0.92	6.08	3.90	2.88	2.40	0.61	0.55

#### Spent Larvae: Indicator Species

Indicator species analysis for the Spent larvae, after deposition onto the pastureland, had notably increased percent abundance differentiating early (weeks 0–5) versus late (weeks 7–19) sampling weeks, as the litter decomposed in the environment (Table 4). Some indicator bacteria were previously discussed in the In-House Larvae indicator species section: *Jeotgalicoccus*, Sphingobacteria, and *Luteimonas*. There are five indicators of the spent larvae that had a higher percent abundance during the early weeks (0–5) in the pastureland.

**TABLE 4:** Indicator species for Spent Larvae sampled while piled in pastureland.

Genera	Indicator Group	PBCC*
*Salinicoccus*	Weeks 0–5	0.636
*Brachybacterium*	Weeks 0–5	0.619
*Jeotgalicoccus*	Weeks 0–5	0.557
Bacillales_unclassified	Weeks 0–5	0.543
*Lactobacillus*	Weeks 0–5	0.525
*Luteimonas*	Weeks 7–19	0.814
*Pseudomonas*	Weeks 7–19	0.778
*Georgenia*	Weeks 7–19	0.748
Rhodobacteraceae_unclassified	Weeks 7–19	0.724
Erythrobacteraceae_unclassified	Weeks 7–19	0.707
Rhizobiales_unclassified	Weeks 7–19	0.706
Flavobacteriaceae_unclassified	Weeks 7–19	0.700
*Halomonas*	Weeks 7–19	0.656
*Devosia*	Weeks 7–19	0.647
*Cellvibrio*	Weeks 7–19	0.633
*Lysobacter*	Weeks 7–19	0.619
Rhizobiaceae_unclassified	Weeks 7–19	0.609
*Paracoccus*	Weeks 7–19	0.600
*Stenotrophomonas*	Weeks 7–19	0.581
Sphingobacteriaceae_unclassified	Weeks 7–19	0.575
*B.42*	Weeks 7–19	0.569
Gemm.3_unclassified	Weeks 7–19	0.535
Alcaligenaceae_unclassified	Weeks 7–19	0.532
*Sphingobacterium*	Weeks 7–19	0.526
*Serpens*	Weeks 7–19	0.513

Samples were grouped in Weeks 0–5 and Weeks 7–19 for comparisons. Only those genera whose relative abundances across all weeks were 0.05% or higher and had a p-value ≤ 0.01 and Point Biserial Correlation Coefficient (PBCC)* value ≥ 0.50 are shown.

*PBCC: Is a correlation between two binary vectors, similar to Indicator values. The higher the PBCC values the stronger the likelihood that a particular taxon is an indicator of that sample.

Most species in the genus *Salinicoccus* have been found in salty environments and are thus described as moderately halotolerant, regulating their osmotic concentrations and thriving at salt concentrations higher than those necessary for growth (Hyun et al., 2013). The salt content in poultry manure tends to be high; therefore, it is environmentally compatible with Salinicoccus bacteria survival from which the larvae may have acquired this bacterium (Li-Xian et al., 2007). *Brachybacterium* were first isolated from deep poultry litter and are considered a harmless environmental bacterium (Collins et al., 1988). They were been isolated from various insects (Zhao et al., 2018; Papadopoulos et al., 2020). Lactobacilli are found in a variety of habitats rich in carbohydrate-containing substrates, including the mucosal, urogenital, and intestinal tracts of animals, as well as material of plant origin, manure, man-made sewage, and spoiling food waste. It produces acetic, propionic, and lactic acids and may reduce the local pH, potentially inhibiting the growth of other bacteria (Slover and Danziger, 2008). Lactobacilli are also normal symbionts of invertebrates (Vásquez et al., 2012).

There were twenty indicators that had a higher percent abundance during the later weeks (7–19) in the pastureland. Pseudomonads are widely distributed and exhibit metabolic diversity. Some species are opportunistic pathogens causing serious illnesses, such as respiratory infections and septicemia, on poultry farms (Kebede, 2010). The *Georgenia* sp. are an alkaliphilic and thermotolerant actinobacterium (Shivlata and Tulasi, 2015). *Devosia* are common in soil habitats and have been found in poultry manure (Lin S. et al., 2021). They can utilize organic sulfur, phosphorus, and aromatic compounds enabling the genus to survive in harsh environments, such as poultry litter (Talwar et al., 2020). Also found in soil, aquatic environments, and poultry litter, the nitrogen-fixing *Cellvibrio* can degrade various polysaccharides, including those in the cell wall of plants (Suarez et al., 2014; Neher et al., 2020). *Lysobacter* have been studied for their production of antifungal agents in the form of lytic enzymes (i.e., chitinases, proteases, and glucanases) (Lin L. et al., 2021). The keratinases produced by this bacterium assist in the degradation of chicken feathers supplementing the decomposition processes within a poultry house (Laba et al., 2018). *Paracocci* are nitrate-reducing bacteria found in the soil and also have diverse degradative capabilities’ such as bioremediation of thiocyanate, halobenzoates, and sulfonates (Baker et al., 1998). Stenotrophomonads are common in soil and plants but can also be opportunistic pathogens (Adegoke et al., 2017). The Methylothermaceae, B42, first identified in deep-sea hydrothermal vents, is a methanotroph capable of denitrification and oxidizing methane, respiring over a wide range of oxygen concentrations (Skennerton et al., 2015). Serpens is closely related to Pseudomonads and is often found in eutrophic aquatic ponds or sewage lagoons as aerobic bacteria in the upper water layers, where it catabolizes lactate, acetate, and pyruvate (Hespell, 1992).

#### Spent Larvae: Relative Abundance at the Phylum Level

For each week after the poultry litter was piled in the pastureland, the relative abundance of bacteria associated with the Spent Larvae fluctuated at the phylum level (Figure 1B). The In-House Larvae had a higher average proportion of Tenericutes (28.7%) across the 11 Flock rotations, followed by Firmicutes (26.5%), Actinobacteria (22.6%), Proteobacteria (18.9%), Bacteroidetes (1.9%), and the then unclassified bacteria (1.4%). Once the litter was piled in the pastureland, then the larvae had a higher average proportion of Proteobacteria (44.4%), followed by Tenericutes (15.7%), Firmicutes (14.8%), Actinobacteria (13.7%), Bacteroidetes (6.7%), and the then unclassified bacteria (3.6%). However, over then the weeks at the pastureland site, the proportion of Proteobacteria fluctuated greatly from a start at 11.5% during Week 0 to a high of 82.2% during Week 15 and then decreased to 22.3% in Week 19. The shift to a higher abundance of Proteobacteria and a lower percent of Firmicutes happened between Weeks 5 and 7 and was maintained through Week 17. This also corresponded to more rainfall in the area after Week 5 and an increase in Proteobacteria and a decrease in Firmicutes in the piled litter itself during this time period (Crippen et al., 2021). It should be noted that LML at the collection site was more readily located with the added moisture to the litter pile (personal observation).

#### Spent Larvae: Beta Diversity and Analysis of Molecular Variance

The NMDS ordination of the Spent Larvae microbial composition shows little in the way of similarities between the microbial profile components over the weeks (Figure 2B). The weighted heat map of the genus level taxa associated with the LML larvae corroborates shifts in microbial genera profiles between the weeks inhabiting the pastureland (Figure 3B). The clustering analyses cluster Native soil, Post Spread, and early Weeks (0–5) together. Within that clade, Native soil clusters into its own clade as having a different microbial community profile from LML. In the next level clades, Week 5 and then the Post Spread samples separate out. The Post Spread consists mostly of soil with litter spread at 0.9MT/0.4 ha. Therefore, the LML had little in common with the pastureland soil microbial community except in Week 5, which showed a distinct increase in Bacteroidetes and unclassified bacteria over the previous weeks. The other major clade groups the later weeks (Weeks 7–19) together.

Based on the heat map results an AMOVA analysis of OTUs at the genus level (*P* ≥ 0.05) reaffirmed that the group of Weeks 1 through 5 is significantly different (*p* = 0.003) than Weeks 7–19. A comparison evaluating which bacteria varied over the weeks that the LML was in the pastureland was conducted by comparing the early weeks (Week 0 through 5) to the later weeks (7 through 19). It is characterized by the increasing mean% abundance (>1% change) between early and late weeks of genera *Pseudomonas*, *Providencia*, *Corynebacterium, Lysobacter*, *Ignatzschineria*, *Escherichia*, and *Halomonas*, and unclassified members of the families Flavobacteriaceae, Sphingobacteriaceae, and Alcaligenaceae, along with class Proteobacteria and the decreasing mean % abundance (>1% change) of *Staphylococcus*, *Atopostipes*, *Brachybacterium*, 5-7N15, *Brevibacterium*, *Yaniella*, *Salinicoccus*, *Solibacillus*, and unclassified members of the families Nocardiopsaceae, Ruminococcaceae, Bacillaceae, and Enterobacteriaceae and orders Clostridiales, Actinomycetales, Entomoplasmatales, along with the phylum Bacteroidetes.

**FIGURE 4:**
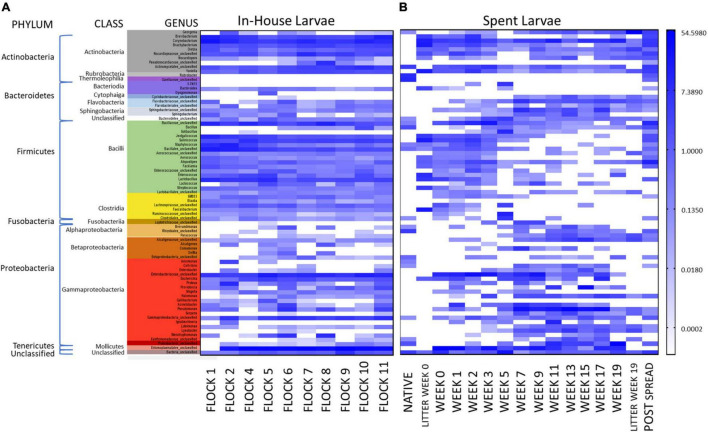
A heatmap of percent abundance of the top 55 identified genera sequences (unweighted) in the LML from the **(A)** In-House (grouped by class) collected during the flock rotations and **(B)** pastureland soil prior to deposition of the spent litter (Native Soil), samples of the spent litter at the time of deposition into the pastureland and just prior to spreading (Litter Week 0 and Litter Week 19, respectively); the soil/litter combination after spreading onto the pastureland (Post Spread); and the larvae collected from that litter over the 19 weeks of stockpiling (Week 0 through Week 19). For natural log transformation “0” was converted to “0.01.”

Additionally, a comparison was conducted to evaluate the differential changes in the bacterial profile once the LML was moved from In-House to the pastureland. The changes in mean % abundance of bacteria associated with the larvae after they were moved to the pastureland showed increases in the genera *Pseudomonas*, *Providencia*, *Acinetobacter*, *Halomonas*, *Lysobacter*, *Ignatzschineria*, *Escherichia* along with an unclassified member of the families Sphingobacteriaceae, Enterobacteriaceae, Alcaligenaceae, Flavobacteriaceae, and Ruminococcaceae. Additionally, the genera which decreased the most were *Brachybacterium*, *Corynebacterium*, *Salinicoccus*, *Jeotgalicoccus*, *Staphylococcus*, *Lactobacillus*, and *Brevibacterium*, along with members of the family Nocardiopsaceae and orders Bacillales and Entomoplasmatales (Figure 4).

It is presumed that the litter environment has a strong influence on the LML microbial community, and in turn, the local environment impacts the piled litter in which the LML resides. The physiological properties of this pastureland litter were reported in a previous publication (Crippen et al., 2016) and demonstrate that Ca, Cu, Fe, Mg, percent moisture, NO_3_^–^/N, % organic matter, pH, P, and Zn all increased; K and Mn increased only slightly; while electrical conductivity, Na, and S decreased in the litter over the weeks it was piled in the pastureland. This could have influenced the bacterial community associated with the LML and reciprocally the larvae could have exchanged bacteria with the litter. Wynants et al. (2018) showed that species-level operational taxonomic units present in the feed in which LML was reared were also detected in the larvae and our lab has also demonstrated the exchange of bacterial pathogens between LML and their environment (Crippen et al., 2018). Such changes in the bacterial community structural changes likely affect the metabolic capabilities of the community associated with the LML. Utilizing the data previously published by our lab (Crippen et al., 2021) on the litter microbial community, a comparison evaluating the abundance of bacteria common to both larvae and spent litter during the time it was piled in pastureland is shown in Figure 4. The litter microbial profile at the time of deposition into the pastureland and just prior to spreading (Litter Week 0 and Week 19, respectively) are included for comparison. However, it should be noted that the microbiome has been shown to be highly variable in the soil environment, differing significantly by just a few centimeters apart (Fierer, 2017). This is presumably true in the litter environment as well, particularly due to abiotic stressors, such as water availability, oxygen concentrations, pH, and carbon availability, which is why composite samples were made in this study (Fierer, 2017). Also, during collection, it was noted that the density of LML within the spent litter fluctuated greatly over the weeks in the pastureland and was much less dense than in In-House populations (personal observation). Therefore, it would require further study to conclude any absolute influence of the spent litter on the LML microbiome.

However, the most numerous bacteria carried by Spent LML showed an increase (>1% change mean % abundance) between Weeks 0 and 19 in the genera *Georgenia*, *Dietzia*, *Lysobacter*, and *Paracoccus*, along with unclassified members of the families Alcaligenaceae and Flavobacteriaceae, the order Rhizobiales, and the class Gammaproteobacteria (Figure 4). Conversely, there was a decrease in the genera *Brevibacterium*, *Jeotgalicoccus*, *Lactobacillus*, *Yaniella*, *Corynebacterium*, *Salinicoccus*, and *Brachybacterium*, along with unclassified members of the families Nocardiopsaceae and Bacillaceae and the order Bacillales.

All microbes (including beneficial and pathogenic bacteria) associated with the LML are important to assess while they are inhabiting the poultry house and when inadvertently transferred in poultry litter used as fertilizer into the environment. In this study, the microbial profile was surprisingly stable while the larvae inhabited the controlled environment within the poultry house, and this, despite changes in the litter microbial profile, indicates their capacity to be a static vessel for some bacterial taxa. More fluctuation in their microbiome occurred over time once the larvae were transferred to the pastureland and exposed to unpredictable environmental influences. Many of the bacterial genera remained with the larvae during their transition from the poultry house into the pastureland; however, in general, the percent of the phyla Firmicutes (classes Bacilli and Clostridia) decreased, along with Tenericutes (class Mollicutes) and Actinobacteria (class Actinobacteria), while that of Proteobacteia (classes Alpha, Beta, and Gammaproteobacteria) and Bacteroidetes (classes Bacteroidetes, Cytophagia, Flavobacteria, and Sphingobacteria) increased slightly. From a risk assessment perspective, the decrease in some Firmicutes could be advantageous. However, while in the litter, these mealworms are producing large amounts of frass and their microbes contribute to the conversion of minerals into a usable form that can boost the fertilizing quality of the litter. The growth in the commercial industry of insect production for protein has led to the assessment of mealworm frass from that production, as a fertilizer. Recent studies have been conducted on the frass of the closely related yellow mealworm (*Tenebrio molitor* L.) and it effectively contributed nitrogen, phosphorus, manganese, magnesium, and potassium that improved plant growth parameters. However, the microbial profile was dependent on the diet of the mealworm and thus likely their microbial gut constituents (Poveda et al., 2019; Houben et al., 2021).

This study is unique in following the microbiome of the LML from its introduction into a brand new broiler facility, through 11 flock rotations, and when deposited in the pastureland with the spent litter where the LML thus becomes accessible to other fauna. Such a longitudinal study has not been previously conducted, nor it had not been shown that the LML can survive months in the spent litter outside of the poultry house. Additionally, the influence of the local environmental microbiome could be assessed and compared due to our previous microbiome and physiological characteristic studies of the surrounding litter and soil conducted simultaneously. However, as with any microbiome study, the shortcomings of the technology utilized (e.g., DNA extraction, sequencing, analytical software, and databases) and the practical limitations of the frequency and breadth of the sampling encumber our full understanding of the microbiome. It should also be noted that this study was conducted at only one broiler house, with their unique environment, feed, and management structure, all of which can influence the resulting local microbiome. With these limitations in mind, however, given the importance of this arthropod within the agricultural industry both as a pest within the stored grain and the poultry industry, as well as an insect approved for development as a possible source of protein within the insect agriculture industry, it is a notably understudied insect. Therefore, this research provides a wealth of information not previously measured.

In conclusion, in this study, we measured the microbial profile of LML from a newly constructed poultry house, through 11 flock rotations and over 19 weeks after the house litter (containing LML) was cleaned out of the poultry house and placed in pastureland. The late instar larvae appeared in the house near the end of the first flock grow-out period and persisted throughout the subsequent 11 flock rotations and at least 19 weeks in pastureland where the spent litter (from Flocks 1 through 9) was deposited. Overall, the main conclusions derived from this study were that, once established, the microbiome of the larvae in the house remained relatively consistent at the genus level despite changes in the litter environment, i.e., partial and total cleanout procedures, the addition of new bedding, and addition of birds. The LML also survived at least 19 weeks in the field once removed from the controlled environment of the poultry house; the larvae were able to bring with them a substantial microbial community into the pastureland. This community then fluctuated in the pastureland with environmental conditions, for example, rainfall appeared to have some influence. Considering the ability of the LML to sustain an established microbial community despite management changes to their poultry house environment and their ability to survive for months within the spent litter in an open field environment, further studies should be done to examine LML efficacy as a reservoir of both beneficial and pathogenic microbial organisms. In particular, beneficial microbial organisms whose metabolic processes contribute minerals to the litter through frass could enhance plant growth when the litter is used as a fertilizer. The characterization of their microbiome is the first step to investigating the influences of their microbes on the manmade and environmental ecosystems.

## Data Availability Statement

The datasets presented in this study can be found in online repositories. The names of the repository/repositories and accession number(s) can be found below: https://www.ebi.ac.uk/ena, PRJEB47987.

## Author Contributions

TC and CS conceived the idea, designed the study, and executed the experiments. BS and TC performed the analyses, bioinformatics, and graphical presentations. TC wrote the manuscript. CS, BS, and RA helped to review and edit the final manuscript. All authors contributed to the article and approved the submitted version.

## Author Disclaimer

Mention of trade names, companies, or commercial products in this publication is solely for the purpose of providing specific scientific information and does not imply recommendation or endorsement of the products by the U.S. Department of Agriculture.

## Conflict of Interest

The authors declare that the research was conducted in the absence of any commercial or financial relationships that could be construed as a potential conflict of interest.

## Publisher’s Note

All claims expressed in this article are solely those of the authors and do not necessarily represent those of their affiliated organizations, or those of the publisher, the editors and the reviewers. Any product that may be evaluated in this article, or claim that may be made by its manufacturer, is not guaranteed or endorsed by the publisher.

## Footnotes

1
https://soilseries.sc.egov.usda.gov/osdname.aspx

2
https://www.merckvetmanual.com/poultry/necrotic-enteritis/overview-of-necrotic-enteritis-in-poultry

3
http://tree.bio.ed.ac.uk/
