# Supplementary material for: Management Practices Affecting Lesser Mealworm Larvae (Alphitobius diaperinus) Associated Microbial Community in a Broiler House and After Relocating With the Litter Into Pastureland
Source: Front Microbiol. 2022 Jul 1;13:875930. doi: 10.3389/fmicb.2022.875930 (PMC9283091; doi:10.3389/fmicb.2022.875930)
Supplement: Supplementary file 1 [file Data_Sheet_1.zip › Supplementary Material/Figure S1.pdf]

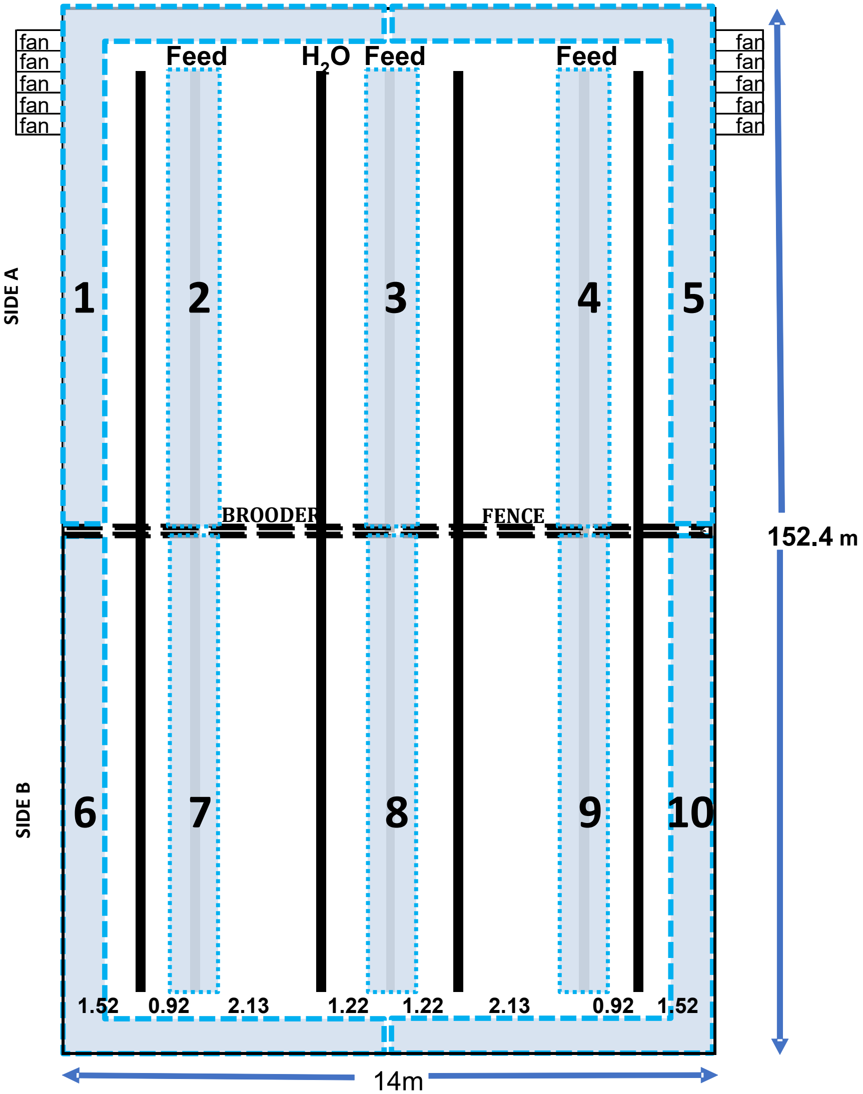

S1 Fig. Schematic of the broiler facility. It was a standard tunnel ventilated metal house, 14 m wide (North/South) by 152.4 m in length (East/West) in size. Alternating water and feed lines ran the length of the house consisting of 4 water (black) and 3 feed (gray) lines. The approximate spacing between the lines is marked in meters (1.52, 0.92, 2.13 and 1.22 m). The first two weeks of each flock rotation, poults were restricted to Side B using a brooder fence; after which the fence was removed, and the bird had full range of the house. Ten sampling areas (shaded blue) are outline consisting of 0.5 m either side of each feed line and 1 m wide along the perimeters of the house for each side A and side B.
