# Supplementary material for: Management Practices Affecting Lesser Mealworm Larvae (Alphitobius diaperinus) Associated Microbial Community in a Broiler House and After Relocating With the Litter Into Pastureland
Source: Front Microbiol. 2022 Jul 1;13:875930. doi: 10.3389/fmicb.2022.875930 (PMC9283091; doi:10.3389/fmicb.2022.875930)
Supplement: Supplementary file 1 [file Data_Sheet_1.zip › Supplementary Material/Figure S2.pdf]

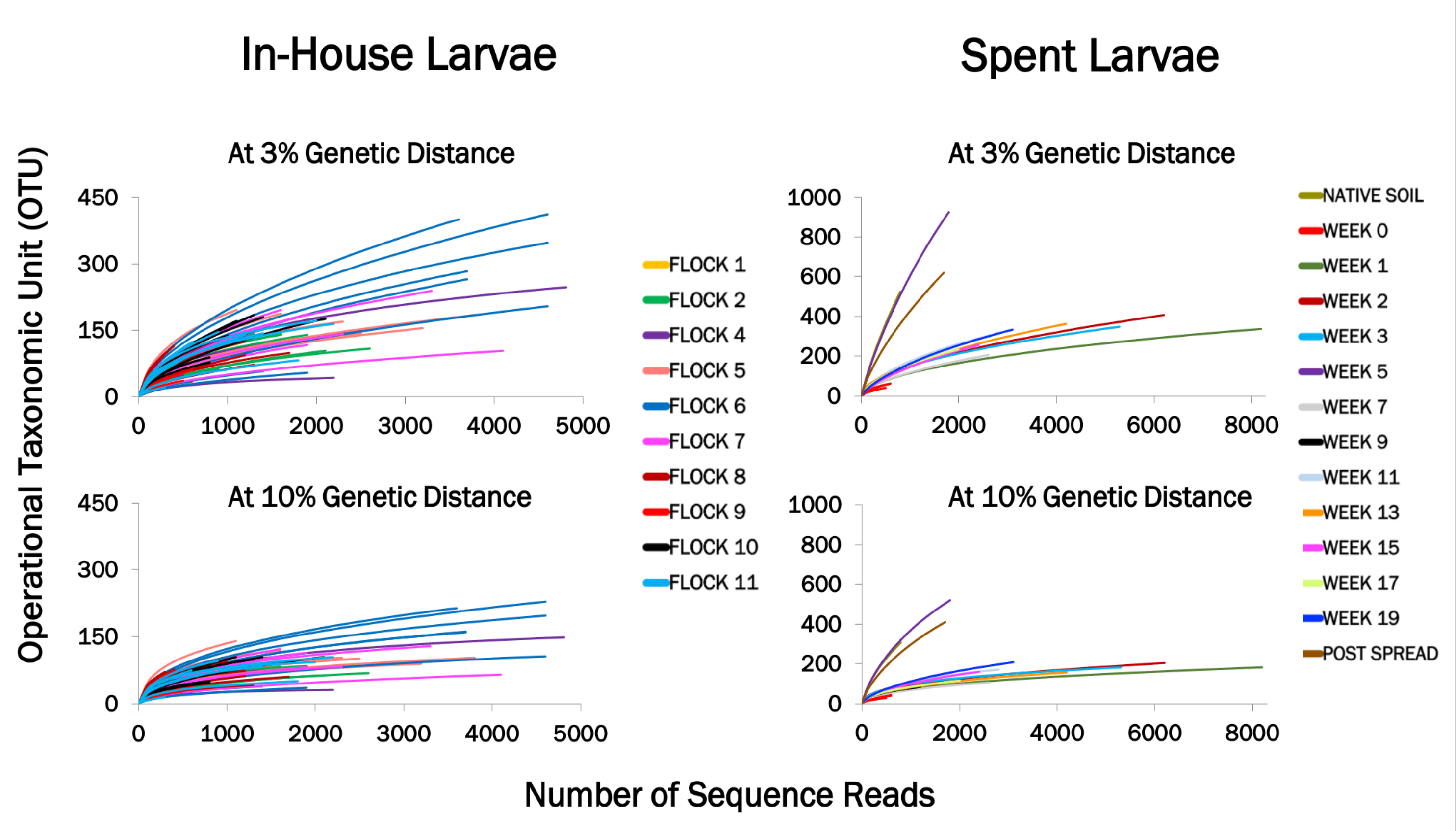

S2 Fig. Rarefaction curves of operational taxonomic units (OTU) at 0.03 and 0.10 genetic distances for A) In-House Larvae and B) Spent Larvae.
