# Supplementary material for: Management Practices Affecting Lesser Mealworm Larvae (Alphitobius diaperinus) Associated Microbial Community in a Broiler House and After Relocating With the Litter Into Pastureland
Source: Front Microbiol. 2022 Jul 1;13:875930. doi: 10.3389/fmicb.2022.875930 (PMC9283091; doi:10.3389/fmicb.2022.875930)
Supplement: Supplementary file 1 [file Data_Sheet_1.zip › Supplementary Material/Figure S3.pdf]

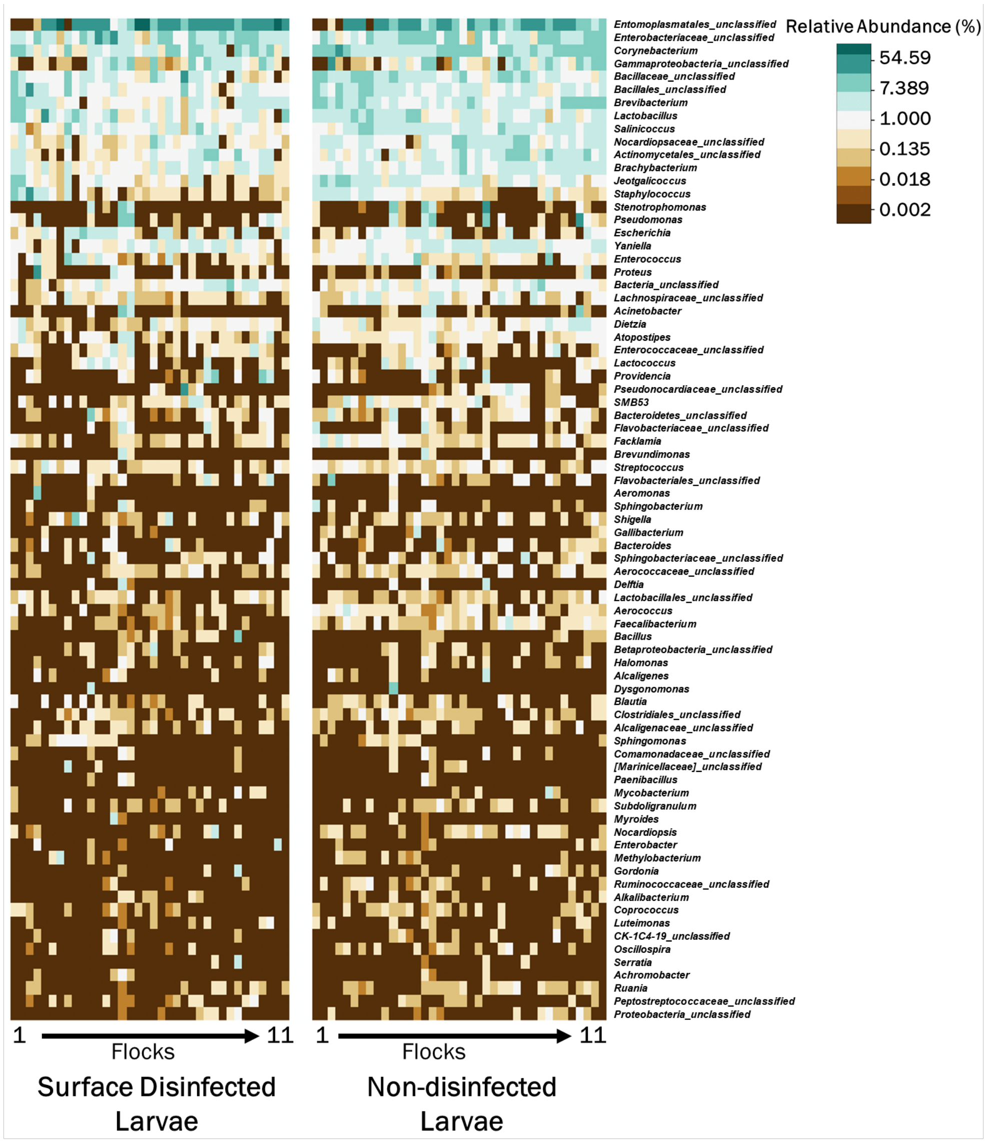

S3 Fig. Heatmap of bacterial genera (total relative abundance across flocks ≥ 0.05), associated with surface disinfected (left) and non-disinfected (right) In-House Larvae. For natural log transformation, “0” was converted to “0.001”.
