# Supplementary material for: Management Practices Affecting Lesser Mealworm Larvae (Alphitobius diaperinus) Associated Microbial Community in a Broiler House and After Relocating With the Litter Into Pastureland
Source: Front Microbiol. 2022 Jul 1;13:875930. doi: 10.3389/fmicb.2022.875930 (PMC9283091; doi:10.3389/fmicb.2022.875930)
Supplement: Supplementary file 1 [file Data_Sheet_1.zip › Supplementary Material/Table S1.pdf]

S1 Table. The feeding regime and basic composition of the ration types fed to the poultry over the grow-out period based on bird age. Bacitracin methylene disalicylate was used as a growth promotor at 50 ppm (50g/ton feed) with the starter, grower and finisher rations. Note: detailed feed composition is proprietary.

Bird Age (days)	Ration	Crude Protein (%)	Vitamin (%)	Trace Mineral (%)	Metabolizable Energy Kcal Kg^{-1}
1-15	Starter	20	100	100	2850
15-28	Grower	19	80	80	2900
29-39	Finisher #1	16	60	60	3000
40-46	Finisher #2[a]	15	40	40	3000
47-50	Withdrawal[b]	15	40	40	3000

[a] If birds are older than 50 days, Finisher #2 is fed for a longer period of time.

[b] Withdrawal ration is fed only for the last 3 days of the grow-out, and is Finisher #2 without additives.
