# Supplementary material for: Management Practices Affecting Lesser Mealworm Larvae (Alphitobius diaperinus) Associated Microbial Community in a Broiler House and After Relocating With the Litter Into Pastureland
Source: Front Microbiol. 2022 Jul 1;13:875930. doi: 10.3389/fmicb.2022.875930 (PMC9283091; doi:10.3389/fmicb.2022.875930)
Supplement: Supplementary file 1 [file Data_Sheet_1.zip › Supplementary Material/Table S3.pdf]

S3 Table. Summary of the percent abundance of the major bacterial phyla in a mixed insect study, the yellow mealworm (YM; *Tenebrio molitor*) and the lesser Mealworm (LM; *Alphitobius diaperinus*) from this and other studies as referenced. The 16s rRNA region targeted by the primer for each study is noted.

	primer region	Actinobacteria	Bacteroidetes	Firmicutes	Fusobacteria	Proteobacteria	Tenericutes	unclassified bacteria	
**mixed insects**	V1-V2	4.8	6.4	20.7	< 1.0	62.1	1.9	3.0	Yun et al. (2014)
**LM**	V1-V3	22.6	1.9	26.5	0.1	18.9	28.7	1.3	In-House Larvae
**LM**	V1-V3	13.7	6.7	14.8	0.5	44.4	15.7	3.6	Spent Larvae
**LM**	V3-V4	8.0	26.0	8.0	<1.0	57.0	nd	<1.0	Cucini et al. (2020)
**LM**	V4	5.0	< 1.0	20.0	nd	68-78	nd	nd	Wynants et al. (2018)
**YM**	V3	0.1	0.1	13.9	3.3	39.2	44.2	< 2.0	Garofalo et al. (2017)
**YM**	V4-V5	26.9	2.9	31.1	nd	35.9	nd	2.5	Stoops et al. (2017)
**YM**	V1-V3	nd	nd	26.2	nd	34.1	36.3	3.1	Jung et al. (2014)

nd = data not reported in the study
